# Adaptive Sparse Representation for Source Localization with Gain/Phase Errors

**DOI:** 10.3390/s110504780

**Published:** 2011-05-02

**Authors:** Ke Sun, Yimin Liu, Huadong Meng, Xiqin Wang

**Affiliations:** Department of Electronic Engineering, Tsinghua University, Beijing 100084, China; E-Mails: yiminliu@tsinghua.edu.cn (Y.L.); menghd@tsinghua.edu.cn (H.M); wangxq_ee@tsinghua.edu.cn (X.W.)

**Keywords:** direction-of-arrival estimation, adaptive sparse representation, adaptive overcomplete basis learning

## Abstract

Sparse representation (SR) algorithms can be implemented for high-resolution direction of arrival (DOA) estimation. Additionally, SR can effectively separate the coherent signal sources because the spectrum estimation is based on the optimization technique, such as the *L*_1_ norm minimization, but not on subspace orthogonality. However, in the actual source localization scenario, an unknown gain/phase error between the array sensors is inevitable. Due to this nonideal factor, the predefined overcomplete basis mismatches the actual array manifold so that the estimation performance is degraded in SR. In this paper, an adaptive SR algorithm is proposed to improve the robustness with respect to the gain/phase error, where the overcomplete basis is dynamically adjusted using multiple snapshots and the sparse solution is adaptively acquired to match with the actual scenario. The simulation results demonstrate the estimation robustness to the gain/phase error using the proposed method.

## 1. Introduction

Direction of arrival (DOA) estimation has long been a useful method for signal detection in sonar, radar and communication applications [1,2]. Subspace-based methods such as minimum variance distortionless response (MVDR) and multiple signal classification (MUSIC) [3,4] require sufficient stationary snapshots to guarantee the high-resolution estimation performance. These methods exploit the orthogonality between the signal and noise subspaces to achieve high-resolution spectrum estimation. In addition, calibration techniques are added to improve the performance in the gain/phase error scenario [1,2]. However, even with appropriate calibration, subspace-based methods are unable to deal with the coherent signal sources because the statistical properties, *i.e.*, the subspace orthogonality cannot provide useful information for separating coherent sources [3,4]. In addition, sufficient snapshots are often unavailable in fast-changing scenarios, which results in inappropriate estimation of the subspaces, and thus, the performance of the DOA estimation is also degraded. Focusing on the problems of coherent sources and the requirements for sufficient stationary snapshots, the sparse representation (SR) method is proposed [5,6]. The key assumption is that the signal sources can be viewed as far-field point sources, and their number is quite small compared with the whole spatial domain. When this assumption is valid, the underlying spatial spectrum is sparse (*i.e.*, has only a few nonzero elements), and we can solve the inverse problem with sparse constraint to approximate the actual sparse signal. Additionally, SR has also been widely used in a variety of other problems, including image reconstruction [7,8], feature selection [9] in machine learning, radar imaging [10,11], and penalized regression [12,13]. In the most basic form, SR attempts to find the sparsest signal **α** satisfying **x** = **Φα**, where **Φ** ∈ *C*^*m*×*n*^ is an overcomplete basis, *i.e.*, *m* ≤ *n* and **x** is the observation data. Without the prior knowledge that **α** is sparse, the equation **x** = **Φα** is ill-posed and has many solutions. Additional information that **α** should be sparse allows one to eliminate this ill-posedness [14–16]. Solving the ill-posed problem involving sparsity typically requires combinatorial optimization, which is intractable even for modest data size. A number of practical algorithms such as convex optimization (including the *L*_1_ norm minimization) [5] and iterative reweighted least squares [6] have been proposed to approximate the actual solution to this problem. However, in the actual array scenario, the unknown gain/phase error between sensors is inevitable. At this case, a mismatch exists between the actual array manifold and the corresponding columns of the predefined basis, which causes performance degradation in DOA estimation [5]. Therefore it is indispensable to design an adaptive SR algorithm where the overcomplete basis is dynamically adjusted to better fit the received data.

In this paper, an adaptive SR algorithm to dynamically adjust both the overcomplete basis and the sparse solution so that the solution can better match the actual scenario is proposed. The remainder of this paper is organized as follows. Section 2 describes the basic model of the received data. In Section 3, an adaptive SR method to deal with the gain/phase error scenario is illustrated. In Section 4, the performance analysis is implemented to illustrate the robustness of adaptive SR with the simulated data. Section 5 presents our concluding remarks about the proposed algorithm.

## 2. Problem Description and Modeling

Source localization using sensor arrays is a problem with important practical applications including radar, sonar, exploration seismology and many other applications [1,2]. In many source localization applications, the physical dimensions of the sources are quite small or the sources are far enough from the array sensors so that they can be viewed as far-field point sources. Although the non-uniform and nonlinear configurations such as conformal array sensors (CFA) have certain advantages over uniform linear array (ULA), in this paper, the discussion is just implemented with the widely-used ULA deployment for simplicity. Next the signal model received by ULA is first given.

### 2.1. Signal Model

**Figure 1.**
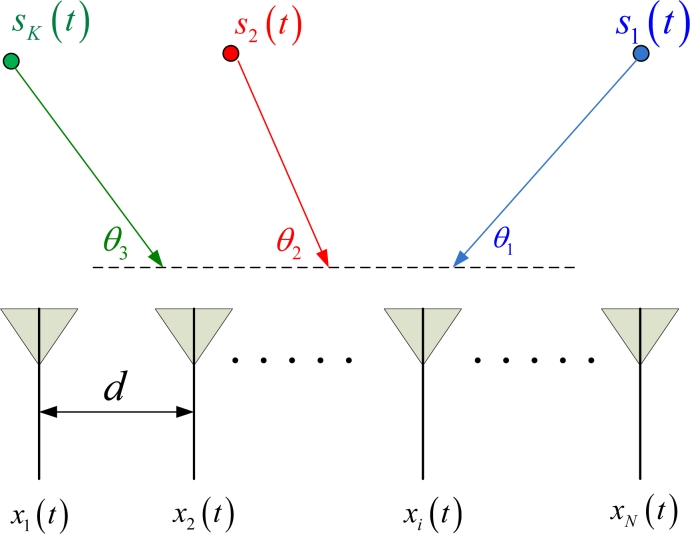
An illustration of the array geometry of source localization.

As shown in Figure 1, the array geometry is assumed to be ULA with *N* sensors, labeled as *x_i_* (*t*), 1 ≤ *i* ≤ *N*, where *t* and *i* indicate the snapshot and sensor indexes, respectively. The inter-sensor spacing was *d*, the radar wavelength is *λ* and the incoming far-field point sources are *s_k_* (*t*), 1 ≤ *k* ≤ *K*, where *K* indicates the number of sources, which is less than the number of sensors.

Starting with the ideal model with no gain/phase error, we have **x** = **Ψs** + **n**, where **x**(*N* × 1) represents one received snapshot, **n**(*N* × 1) is the noise vector, **s**(*K* × 1) is the source vector, and the matrix **Ψ**(*N* × *K*) is the steering vectors of the actual sources, *i.e.*, the array manifold as [1,2]:

(1) $$\Psi =\left[\begin{array}{cccc} \text{exp}\left(\frac{d}{\lambda }\text{sin}\;\theta_{1}\cdot 0\right), & \text{exp}\left(\frac{d}{\lambda }\text{sin}\;\theta_{2}\cdot 0\right), & \cdots & \text{exp}\left(\frac{d}{\lambda }\text{sin}\;\theta_{K}\cdot 0\right)\\ \text{exp}\left(\frac{d}{\lambda }\text{sin}\;\theta_{1}\cdot 1\right), & \text{exp}\left(\frac{d}{\lambda }\text{sin}\;\theta_{2}\cdot 1\right), & \cdots & \text{exp}\left(\frac{d}{\lambda }\text{sin}\;\theta_{K}\cdot 1\right)\\ \vdots & \vdots & \cdots & \vdots \\ \text{exp}\left(\frac{d}{\lambda }\text{sin}\;\theta_{1}\cdot (N\;-1)\right), & \text{exp}\left(\frac{d}{\lambda }\text{sin}\;\theta_{2}\cdot (N\;-1)\right), & \cdots & \text{exp}\left(\frac{d}{\lambda }\text{sin}\;\theta_{K}\cdot (N\;-1)\right) \end{array}\right],$$

where the vector:

(2) $$s\;(\theta_{k})\;=\;\left[\text{exp}\left(\frac{d}{\lambda }\;\text{sin}\;\theta_{k}\;\cdot \;0\right),\;\text{exp}\left(\frac{d}{\lambda }\;\text{sin}\;\theta_{k}\;\cdot \;1\right),\;\cdots \text{exp}\left(\frac{d}{\lambda }\;\text{sin}\;\theta_{k}\;\cdot \;(N\;-\;1)\right)\right],$$

indicates the steering vector of the actual source with angle *θ_k_*. Once the actual array manifold **Ψ** is known, the technique of data fitting can be used to estimate the signal amplitudes of the actual sources [1,2]. However, in the actual radar array environment, the actual manifold is unavailable and needs to be estimated. To avoid this problem, we deign an overcomplete basis containing all the steering vectors. Then the spectrum estimation can be implemented by solving the underdetermined equation instead of finding the actual array manifold. Discretize the angle axis into *N_s_* = *ρ_s_N* (*ρ_s_* ≫ 1) grids so that $\phi_{i}\;=\;\frac{2\pi i}{N_{s}},\;1\;\leq \;i\;\leq \;N_{s}$ denotes the uniformly-discretized angles. Then the *N* × *N_s_* overcomplete basis is given as [5]:

(3) $$\Phi \;=\;\left[s(\phi_{1}),\;\cdots ,\;s(\phi_{2}),\;\cdots ,\;s\left(\phi_{N_{s}}\right)\right],$$

where **s** (*ϕ_i_*) is the steering vector corresponding to angle *ϕ_i_*. Then the snapshot **x** can be rewritten in matrix form as:

(4) x = Φα + n,

where **α**(*N_s_* × 1) represents for the actual spectral distribution. The actual array manifold **Ψ** corresponds to the steering vectors of the significant elements in **α**, and ideally, is the subset of the overcomplete basis **Φ**. Therefore finding the actual array manifold is equal to picking up the corresponding columns from the overcomplete basis. Because *N_s_* > *N*, the underdetermined problem of solving **α** in (4) is generally ill-posed. Prior works have illustrated that with the additional information that the spatial spectrum, *i.e.*, the solution **α** is sparse, this ill-posedness can be effectively removed [5,6]. Solving problems involving sparsity typically requires combinatorial optimization, which is intractable even for modest data sizes, therefore, a number of approximations have been considered [14,15]. Next we give a brief synopsis of relevant ideas in sparse representation.

### 2.2. Sparse Representation

Recently, the techniques of SR have been illustrated as effective methods for DOA estimation [5,6]. The SR technique, by its nature, can separate coherent sources because the spectrum estimation is based on the optimization technique, but not on subspace orthogonality. Moreover, when multiple stationary snapshots are available, further improvements on estimation performance are expected with the “joint-sparse” characteristic [5,6].

#### 2.2.1. Single Snapshot Case

With the constraint of sparsity on **α** (only a small subset is nonzero), the problem in (4) can be efficiently solved by SR [5] as:

(5) $$\hat{\alpha }\;=\;\text{arg}\;\text{min}||\alpha {||}_{1}\;\text{subject}\;\text{to}\;||x\;-\;\Phi \alpha {||}_{2}\;\leq \;ɛ,$$

where ‖·‖*_p_* stands for the *L_p_* norm and ɛ is the error allowance in sparse representation. During the optimization, the *L*_2_ norm constraint by ɛ guarantees the residual ‖**x** – **Φα**‖_2_ to be small, whereas the *L*_1_ norm enforces the sparsity of the estimated spectrum **α**. In fact, the exact sparsity, *i.e.*, the number of the nonzero elements should be originally given by the *L*_0_ norm. However, this optimization is NP-hard and is unrealizable even for modest data size [14,15]. Unlike the *L*_0_ norm, the *L*_1_ norm minimization can be efficiently implemented via convex optimization. The fundamental contribution of SR is to illustrate the equivalence between these two optimizations. It is proven that SR implemented by the *L*_1_ norm minimization can approximate the actual solution as ‖**α̂** – **α**_0_‖_2_ ≤ Λ · ɛ, where **α_0_** indicates the actual sparse solution, Λ is the stability coefficient related to the maximal mutual coherence in the matrix **Φ** [15]. The detailed illustration of the *L*_1_ norm characteristic is given in [16]. Therefore SR has the ability of high-resolution estimation. Furthermore, when several stationary snapshots are available, we can combine these snapshots to improve the estimation performance.

#### 2.2.2. Multiple Snapshots Case

When multiple measurements are available, the data model is extended as:

(6) X = ΦS + N,

where **X** = [**x**^(1)^,⋯,**x**^(*L*)^] are multiple snapshots, **N** = [**n**^(1)^,⋯,**n**^(*L*)^] and **S** = [**α**^(1)^,⋯,**α**^(*L*)^] are the corresponding noise and spectrum matrixes, respectively. The rows of **S** indicate the spatial dimension and the columns indicate the temporal dimension. One natural approach using multiple snapshots is to exploit the joint sparse representation characteristic, which assumes that the positions of the significant sources keep identical among different snapshots and the difference is only reflected on their amplitude variations. Chen *et al.* proposed the mixed *L*_1,2_ norm minimization to implement the joint optimization [17–19].

The mixed *L*_1,2_ norm minimization is implemented on the solution matrix **S**, with the definition as $1/L\cdot \sum_{j=1}^{L}{\left(\sum_{i=1}^{N}\left|S_{i,j}\right|\right)}^{2}$.

Based on this, the *L*_1,2_ norm minimization combines the multiple snapshots using the *L*_2_ norm and the sparsity is only enforced in the spatial dimension via the *L*_1_ norm. Therefore the solution matrix **S** is parameterized temporally and spatially, but the sparse constraint has only been enforced in spatial dimension because the signal is not generally sparse in temporal domain. However, this joint optimization is quite complicate and has a huge computation load. When the number of the snapshots *L* increases, the required computational effort increases superlinearly. Therefore, when the number of the snapshots is large, this approach is not practical for real-time source localization.

##### A. Noncoherent Average

To decrease the computation load, a simple method is to separate the joint problem in (6) into a series of independent subproblems [5] as:

(7) $$x^{\left(l\right)}\;=\;\Psi \alpha^{\left(l\right)}\;+\;n^{\left(l\right)},\;1\;\leq \;l\;\leq \;L.$$

Each subproblem can be solved via the *L*_1_ norm minimization using (5) to obtain the sparse spectrum estimation. Then, the average result of these estimated spectrums **α̂**^(*l*)^, 1 ≤ *l* ≤ *L* can be taken as:

(8) $$\hat{\alpha }\;=\;\frac{1}{L}\;\sum_{l=1}^{L}\left|{\hat{\alpha }}^{\left(l\right)}\right|.$$

This method implements noncoherent average and its main attraction is its simplicity. However, by turning to fully coherent combined processing, as described in the following sections, we expect to achieve greater accuracy and robustness to noise.

##### B. L1-SVD

A typical coherent sparse representation algorithm using multiple snapshots is the ℓ_1_-*SVD* method [5,20]. It implements the sparse estimation only in the signal subspace, and thus, the robustness to noise is improved and the computation load of the optimization is quite low. In its basic form, the received data is decomposed into the signal and noise subspaces using the singular value decomposition (SVD) of the *N* × *L* data matrix **X**. Then the spectrum estimation is molded with reduced dimension only in the signal subspace. Mathematically, this translates into the following representation. Take SVD of the data matrix as:

(9) $$X\;=\;ULV^{H},$$

where the diagonal entries of **L** indicate the singular values of **X**, the columns of **U** and **V** are left- and right-singular vectors, respectively. Suppose the number of actual sources is *K* (*K* ≪ *L*), the reduced dimension *N* × *K* matrix denotes the signal subspace as **X***_SV_* = **ULD***_K_* = **XVD***_K_*, where **D***_K_* = [**I***_K_*, **0**]. Obtain **S***_SV_* = **SVD***_K_* and **N***_SV_* = **NVD***_K_* similarly, and then, the data can be molded in the signal subspace as:

(10) $$X_{SV}\;=\;AS_{SV}\;+N_{SV}.$$

Then the *L*_1_ norm minimization can be similarly implemented like (5), however, only in the signal subspace. In the ℓ_1_-*SVD* method, the noise level is reduced and the spectrum estimation is improved. In addition, the size of the joint optimization is reduced from *N* × *L* into *N* × *K*, and thus, the computation load is greatly reduced. The simulation results in [5] illustrate that ℓ_1_-*SVD* has the advantages of both lower computation load and more robustness to the noise. Therefore, in the simulation part of this paper, the ℓ_1_-*SVD* method is chosen as a performance reference.

However, there are some nonideal factors, which is inevitable in a practical radar array system. These factors include gain/phase error, mutual coupling between sensors and so forth [1,2]. When these happen, the predefined overcomplete basis in SR cannot effectively express the actual array manifold, which causes performance degradation in spectrum estimation. Similar problems also appear in other spectrum estimation methods like MVDR and MUSIC [3,4]. In this paper, we only focus on the gain/phase error scenario and propose an effective method to adaptively calibrate the overcomplete basis so that the robustness of the spectrum estimation is improved. Similar treatments can also be made to deal with the mutual coupling scenario, however, the optimization procedure is more complicate. Without considering the mutual coupling between sensors, the error matrix can be given as [21–23]:

(11) $$\Gamma \;=\;\text{diag}\;\left(\Delta a_{1}e^{j\Delta \theta_{1}},\cdots ,\;\Delta a_{N}e^{j\Delta \theta_{N}}\right),$$

where Δ*a_i_e*^*jΔθ*_i_^ indicates the gain/phase error at the *ith* sensor. In this scenario, the data model is correspondingly modified as:

(12) $$x\;=\;\Gamma \Psi s\;+\;n\;=\;\Psi_{m}s\;+\;n,$$

where **Ψ***_m_* = **ΓΨ** denotes the actual array manifold with the gain/phase error. In SR, the overcomplete basis **Φ** is constructed without considering the gain/phase error since the error matrix **Γ** is unknown in advance. The mismatch exists between **Ψ***_m_* and the corresponding columns of the predefined basis **Φ**, and thus, the estimation performance is degraded. Concerning the ℓ_1_-*SVD* algorithm, the mismatch caused by the gain/phase error still exists in the signal subspace so that the degraded performance is inevitable. Focused on this, an adaptive SR algorithm is proposed in this paper, which dynamically calibrates the overcomplete basis so that the sparse solution can better fit the actual scenario.

## 3. Adaptive Sparse Representation

The key feature of adaptive SR is the adaptive adjustment of the overcomplete basis. This process generally learns the uncertainty of the overcomplete basis, which is not available from the prior knowledge, but rather has to be estimated using multiple snapshots. Prior works on basis learning take the strategy that the whole overcomplete basis is optimized to better represent the data of multiple snapshots [24–26]. However, this optimization has to solve a large amount of variables, *i.e.*, all the elements in the overcomplete basis, and thus, the computation load is quite large. Furthermore, the optimization may deviates from the actual solution because no knowledge is added to guarantee the structure in the basis estimation. In this paper, when only gain/phase error is considered, the unknown error matrix **Γ** is a diagonal matrix [21–23]. Then the actual overcomplete basis has specific structure and can be decomposed into two parts: one is the predefined overcomplete basis **Φ**, the other is the unknown error matrix **Γ**. Therefore, the estimation of the actual overcomplete basis can be implemented only in the error matrix part, where the number of the variables to be solved is greatly reduced and the estimated basis is more robust. In addition, in the presence of gain/phase error, the spectrum estimation in SR is degraded, reflected as spurious peaks and missing of small actual sources. When this error is small or moderate, the positions of the estimated significant sources are still reliable [5,6]. Therefore the steering vectors corresponding to the significant sources in the spectrum estimation of SR can still be served as an effective approximation of the original array manifold **Ψ**. With the aid of the multiple received snapshots, the covariance matrix estimation is obtained as:

(13) $$\hat{R}\;=\;\frac{1}{L}\;\sum_{l=1}^{L}x_{l}x_{l}^{H},$$

where *L* is the number of the snapshots, **x***_l_* is the *lth* snapshot. Then the signal and noise subspaces can be effectively obtained using EVD of the covariance matrix estimation as:

(14) $$\hat{R}\;=\;U\Lambda U^{H},$$

where **U** = [**u**_1_,⋯,**u***_N_*] are the eigenvectors corresponding to the eigenvalues *λ_i_*, 1 ≤ *i* ≤ *N*. Suppose the number of the actual sources is *K*, and the eigenvalues *λ_i_*, 1 ≤ *i* ≤ *N* is sorted in the descending order, the signal subspace is represented as **U***_s_* = [**u**_1_,⋯,**u***_K_*] and the noise subspace is given as **U***_n_* = [**u***_K_*_+1_, ⋯ **u***_N_*]. The signal subspace provides a range space of the actual array manifold **ΓΨ**, *i.e.*, *span* {**U***_s_*} = *span* {**ΓΨ**}[21]. Furthermore, with the orthogonality between the signal and noise subspaces, we have:

(15) $$\text{span}\;(\Gamma \Psi )\;⊥\;U_{n}.$$

Once reliable estimations of **Ψ** and **U***_n_* can be obtained, a reasonable estimate **Γ̂** is given by minimizing:

(16) $$\hat{\Gamma }\;=\;\underset{\Gamma }{\text{min}}\left({\sum_{k=1}^{K}\left\|{\hat{U}}_{n}^{H}\Gamma {\hat{\Psi }}_{k}\right\|}^{2}\right)\;=\;\underset{\Gamma }{\text{min}}\;\left(\sum_{k=1}^{K}{\hat{\Psi }}_{k}^{H}\;\Gamma^{H}\;{\hat{U}}_{n}U_{n}^{H}\Gamma {\hat{\Psi }}_{k}\right),$$

where the matrix **Ψ̂***_k_*, 1 ≤ *k* ≤ *K* indicates the manifold estimation, *i.e.*, the columns corresponding to the significant elements in the spectrum estimation using SR. Additionally, the noise subspace estimation **Û***_n_* can also be obtained using EVD. With the aid of these estimations, the error matrix estimation **Γ̂** can be effectively given using (16). Even though some small sources might be not included in the array manifold estimation, the subspace orthogonality is still valid between the subspace of the significant sources and the noise subspace, and thus, the solution in (16) still serves as an effective approximation of the error matrix. Although the above optimization is well-defined, the corresponding optimization is rather complicate and difficult to implement. Next, simplification is implemented to further improve this optimization process. Define that:

(17) $$\Gamma {\hat{\Psi }}_{k}=a_{k}\delta ,$$

where **a***_k_* is a diagonal matrix given by:

(18) $$a_{k}\;=\;\text{diag}\;\left\{{\hat{\Psi }}_{k}\right\},$$

and **δ** is a vector given by:

(19) $$\delta \;=\;{\left[\Gamma_{11},\;\Gamma_{22},\;\cdots ,\;\Gamma_{MM}\right]}^{T},$$

where Γ*_ij_* indicates the element located at the *ith* row and the *jth* column of matrix **Γ**. Then the minimization in (16) can be rewritten as:

(20) $$\hat{\Gamma }\;=\;\underset{\Gamma }{\text{min}}\;\delta^{H}\;\left\{\sum_{k=1}^{K}a_{k}^{H}{\hat{U}}_{n}{\hat{U}}_{n}^{H}a_{k}\right\}\delta .$$

Similarly, we need to minimize (20) with respect to **δ** under the energy constraint **δ***^H^* **w** = 1, where **w** = [1,⋯,1]*^T^*. The result of this problem is well solved using quadratic optimization and is given by:

(21) $$\delta \;=\;Q^{-1}w/\left(w^{H}\;Q^{-1}w\right),$$

where the matrix **Q** is given as:

(22) $$Q\;=\;\sum_{k=1}^{K}a_{k}^{H}{\hat{U}}_{n}{\hat{U}}_{n}^{H}a_{k}.$$

Then the error matrix estimation can be effectively given as **Γ** = *diag* (**δ**). Unlike (16), the matrix **Q** can be calculated in advance, and thus, the optimization in Equations (21,22) can be directly implemented. The detailed procedures of the adaptive SR algorithm are given as follows:

1. Let *n* = 1 and set the initial error matrix as **Γ̂**^(0)^ = **I**.
2. Calculate the covariance matrix estimation using (13) and obtain the noise subspace as **U***_n_* = [**u**_*K*+1_,⋯**u***_N_*] using SVD.
3. At the *nth* iteration, the sparse solution **α̂**^(*n*)^ is estimated by the *L*_1_ norm minimization with the overcomplete basis **Γ̂**^(*n*−1)^**Φ** as:
  (23) $${\hat{\alpha }}^{\left(n\right)}\;=\;\text{arg}\;\text{min}||\alpha {||}_{1}\;\text{subject}\;\text{to}\;||x\;-\;{\hat{\Gamma }}^{\left(n-1\right)}\Phi \alpha {||}_{2}\;\leq \;ɛ.$$
  where *ɛ* represents a small matching allowance. This optimization can be effectively solved by convex optimization or other approximation algorithms [27]. Then if the solution is converged as $\left|\frac{{\hat{\alpha }}^{\left(n\right)}\;-{\hat{\alpha }}^{\left(n-1\right)}\;}{{\hat{\alpha }}^{\left(n\right)}}\right|\;\leq \;\varsigma$, where *ζ* is a small constant, end the iteration process, otherwise, continue to steps 4–5.
4. Based on the current solution **α̂**^(*n*)^, only significant peaks (local maxima) are extracted from the spectrum estimation and the manifold estimation is given as **Ψ̂** = [**s**(ϕ_*p*_1__),⋯, **s**(ϕ_*p*_*K*__)], where *K* is the number of the extracted peaks, and *p_K_* represents the corresponding column indexes.
5. Update the error matrix using the optimization in Equations (21,22). Then the *n* + 1 iteration is implemented as steps 3–5.

In the adaptive SR, the choice of *K* is quite important because either adding spurious peaks or missing actual sources may cause a subspace deviation and this impacts the estimation performance of the error matrix. Although the *K* value is generally unknown in the actual array scenario, there are several effective methods, such as the Akaike information criterion (AIC) or minimum description length (MDL) for estimating it [28,29]. Therefore, even if *K* is unknown, we can still obtain estimation of the signal subspace by only extracting the subspaces corresponding to the significant eigenvalues. The detailed process of estimating *K* is not discussed in this paper.

## 4. Simulation Result

### 4.1. Robustness to Gain/Phase Error

**Figure 2.**
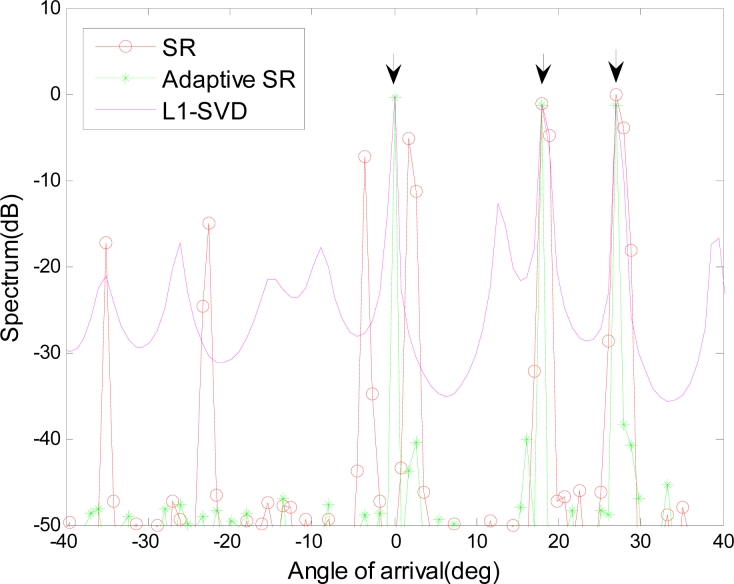
Spectrum estimation result.

In our simulations, a ULA with *N* = 20 sensors is deployed. The inter-sensor spacing is half-wavelength and three far-field sources coming from angles 0°, 18°, 27° are considered. The number of the snapshots is *L* = 20, and the error matrix is given as **Γ** = *diag* (Δ*a*_1_*e*^*j*Δ*θ*_1_^,⋯,Δ*a_N_e*^*j*Δ*θ*_*N*_^), where the gain error obeys Δ*a_i_* ∼ *N*(1,1e^−3^) and the phase error Δ*θ_i_* is uniformly distributed between (−2°,2°). Here, SR implemented the *L*_1_ norm minimization at each snapshot separately and then averages them to obtain the overall performance. The *L*_1_ – *SVD* algorithm is also introduced in this part, which utilizes multiple snapshots and implements the *L*_1_ norm minimization only on the signal subspace [5]. Figure 2 gives the spectrum estimation using different methods, where the arrows indicate the positions of the actual sources. In SR, a high-resolution spectrum is expected, but it is not robust to the gain/phase error and contains spurious peaks. *L*_1_ – *SVD* can reduce the impact of the gain/phase error to some extent, but the performance is limited because the gain/phase error is still inevitable in the signal subspace. The proposed adaptive SR estimates the error matrix and adjusts the overcomplete basis. Therefore it can better match the received snapshot and owns higher estimation accuracy.

**Figure 3.**
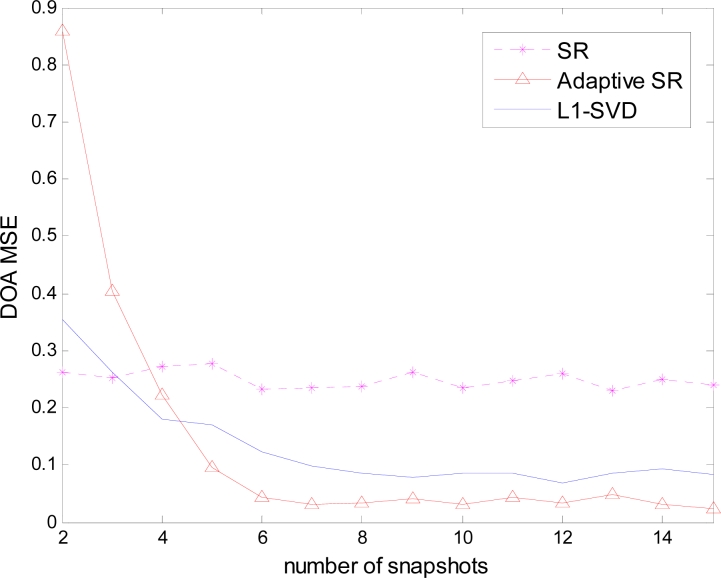
DOA MSE against the number of snapshots.

Next, the quantitative results are given to illustrate the advantages of adaptive SR. All the performance comparisons are based on 50 Monte Carlo simulations. Figure 3 depicts the mean square error (MSE) of the DOA estimation against the number of snapshots, where only peaks are extracted to evaluate the position accuracy.

Since SR deals with each snapshot separately, the addition of snapshots provides no obvious benefits for improving the performance. The performance of *L*_1_ – *SVD* does improve with the adding of snapshots. However, there are spurious peaks because the signal subspace still contains the gain/phase error. In adaptive SR, when the snapshots are not sufficient, *i.e.*, *L* ≤ 4, the manifold estimation **Ψ̂** is not accurate. At this case, adaptive SR cannot effectively express the range space of the actual sources and results in a large MSE. However, the performance of adaptive SR does improve with the addition of the snapshots and is better than the other two methods when the number of snapshots is relatively sufficient (*L* ≥ 6).

**Figure 4.**
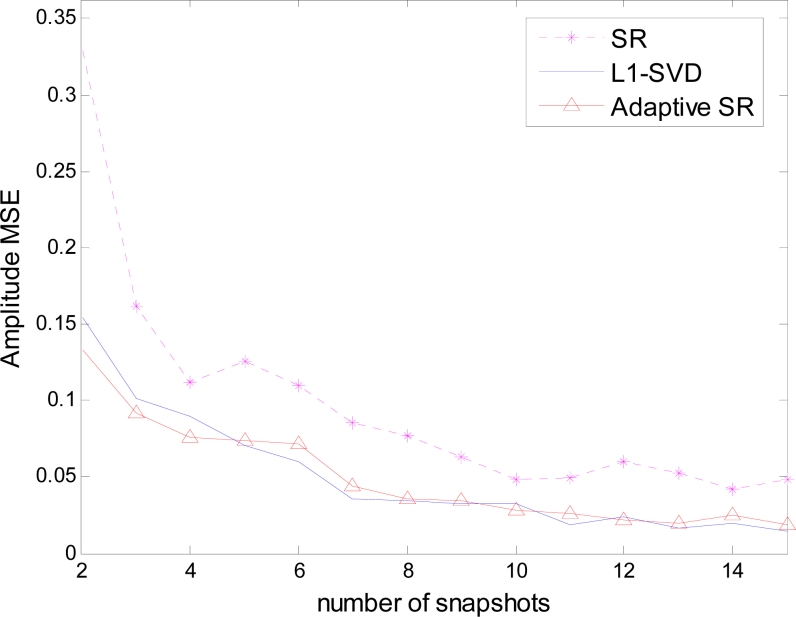
Amplitude MSE against the number of snapshots.

Figure 4 depicts the amplitude MSE against the number of snapshots, where the amplitude is only evaluated on the actual source positions. In this case, both adaptive SR and *L*_1_ – *SVD* can achieve desirable amplitude estimation, which is better than SR. Because the estimation performance includes both the position and amplitude accuracy, the estimation evaluation should be considered including both Figures 3 and 4. In this sense, adaptive SR is better than *L*_1_ – *SVD* in the gain/phase error scenario.

### 4.2. Coherent Sources

**Figure 5.**
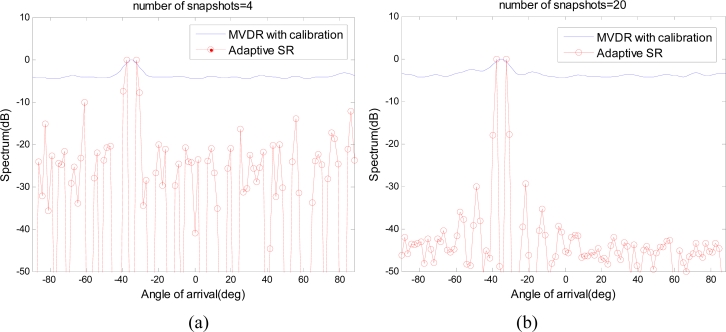
**(a)** Spectrum estimation result with *L* = 4 snapshots. **(b)** Spectrum estimation result with *L* = 20 snapshots.

As stated above, compared with traditional SR methods like *L*_1_ – *SVD*, adaptive SR can significantly improve the estimation robustness of the gain/phase error. In addition, compared with subspace-based methods with calibration [1,21], adaptive SR can deal with coherent signal sources because the final spectrum estimation is still based on the *L*_1_ norm minimization, but not on subspace orthogonality. The following scenario is used to prove the capabilities of dealing with coherent sources of adaptive SR, where subspace-based methods with calibration are ineffective. The array parameters and the gain/phase error keep identical with that in Section 4.1. Two far-field point sources are located at angles −38°, −32°, having a high correlation of *ρ*_12_ = 0.95. As a performance comparison, MVDR is deployed as the detailed implementation of the subspace-based methods [1]. To improve the robustness to the gain/phase error, the array calibration is also employed. The detailed implement of calibration technique is given in [21,22]. As shown in Figure 5(a), when there are insufficient snapshots (*L* = 4) to obtain the statistical properties, the estimation of gain and phase error is not accurate and the calibration performance is limited for both MVDR and adaptive SR. At this case, the overcomplete basis mismatches with the actual array manifold and adaptive SR contains many spurious peaks. Adding more snapshots (*L* = 20) does help to estimate the gain/phase error matrix to calibrate the array sensors, however, the statistical properties dose not improve the estimation performance of MVDR. On the other hand, when adaptive SR has sufficient snapshots to estimate the error matrix, the spectrum estimation can be implemented with a more matched overcomplete basis. Based on this, the estimation performance is improved using *L*_1_ norm minimization and the coherent sources can be effectively separated. Therefore, even though effective calibration is deployed in MVDR, it still can not distinguish the coherent sources. On the other hand, adaptive SR can make it because the final spectrum estimation is still based on *L*_1_ norm minimization, but not on subspace orthogonality.

## 5. Conclusions

This paper focuses on improving the robustness of sparse representation for the DOA estimation with the gain/phase error. By dynamically calibrating the overcomplete basis and adaptively estimating the sparse solution, the proposed adaptive SR can greatly improve the estimation robustness, and thus, the solution better matches the actual scenario. Additionally, it does separate the coherent sources, which is unrealizable for subspace-based methods with calibration. The following are several considerations for further research: first, the current signal model in SR only considers the far-field point sources, however, the near-field source location is also important and meaningful in the actual scenario. Second, the convergence of the adaptive SR needs to be proved in a strict mathematical way. Finally, more adaptive mechanisms should be added to deal with the mutual coupling scenario.
